# Association of morning blood pressure surge with chronic kidney disease progression in patients with chronic kidney disease and hypertension

**DOI:** 10.1111/jch.14366

**Published:** 2021-09-09

**Authors:** Xiang Liu, Ting Zhang, Aiya Qin, Fangming Li, Zhiyao Zheng, Huan Zhou, Yi Tang, Wei Qin

**Affiliations:** ^1^ Division of Nephrology Department of Medicine West China Hospital Sichuan University Chengdu Sichuan China; ^2^ West China School of Medicine Sichuan University Chengdu Sichuan China; ^3^ Division of Nephrology Department of Medicine Chengdu Seventh People's Hospital Chengdu Sichuan China

**Keywords:** ambulatory blood pressure monitoring, cardiovascular events, chronic kidney disease, CKD progression, MBPS

## Abstract

Blood pressure (BP) usually rise from being asleep to awake, which is named the morning blood pressure surge (MBPS). Researches have reported that elevated MBPS was related with CV events, incident CKD in hypertensive patients. However, there have been no studies that have investigated the association between MBPS and renal or heart outcomes in patients with CKD and hypertension, in these patients, the MBPS is much lower because of high prevalence of night hypertension and reduced BP dipping. In this prospective two‐center observational study, we enrolled patients with CKD and hypertension and the 24 h ambulatory blood pressure monitoring (ABPM) was conducted in all patients. Time to total mortality, CKD progression and CV events was recorded; Finally, a total of 304 patients were enrolled and 94 (30.9%) of them had elevated MBPS. After a follow‐up for median 30 months, 23 (7.6%), 34 (11.2%), and 95 (31.3%) patients occurred death, CKD progression and new‐onset CV events, respectively. The Cox regression analysis suggested the elevated MBPS was a strong predictor of CKD progression (HR 2.35, 95%CI 1.2 ‐4.63, *p* = .013), independent of morning BP, while no associations were found between elevated MBPS and CV events (HR 1.02, 95%CI 0.66 ‐1.57), as well as death (HR 1.08, 95%CI 0.46 ‐2.55). In conclusion, we provided the first evidence that elevated MBPS was an important risk factor of CKD progression in patients with CKD and hypertension. Appropriate evaluation and management of MBPS may be helpful to postpone CKD progression.

## 1 INTRODUCTION

Chronic kidney disease (CKD) is considered a major public health issue with a 8–16% prevalence worldwide.^1^, ^2^ Hypertension, as both an important risk factor and a complication of CKD, is very common in CKD patients; the prevalence ranges from 60% to 90% depending on the cause and severity of CKD.^3^, ^4^ Poor control of hypertension increases the risk of development and progression of kidney disease, resulting in end‐stage renal disease (ESRD), cardiovascular (CV) events, and even mortality.^5^, ^6^, ^7^ Ambulatory blood pressure monitoring (ABPM) is an automatic and non‐invasive instrumentation which constitutes a method of BP assessment. The ABPM can provide not only the BP data throughout the day but also the BP variability, BP circadian pattern, etc.^8^ BP circadian pattern is generally characterized by a fall during sleep and a surge in the morning, a rise in BP from being asleep to awake is named the MBPS, which can be calculated from ABPM data.^9^, ^10^

As reported in previous studies, elevated MBPS was associated with CV events in patients with hypertension.^11^, ^12^, ^13^ Furthermore, some researchers have found that the elevated MBPS was a risk factor for incident CKD and kidney function deterioration. A cross‐sectional study enrolling 823 CKD patients showed that elevated MBPS was an independent risk factor for poor renal function, independent of a non‐dipping pattern, and BP level^14^; another prospective study confirmed that elevated MBPS was associated with incident CKD and decline in estimated glomerular filtration rate (eGFR) in hypertensive patients.^15^ However, to best of our knowledge, no studies have addressed the predictive value of MBPS to CV events and CKD progression in patients with hypertension and CKD. In this population, patients had high prevalence of night hypertension and non‐dipping BP,^16^, ^17^ which may result in a small MBPS. Under this situation, it is unclear if MBPS is associated with an increased risk for CV events and CKD progression.

To investigate the relationship between MBPS and prognosis in CKD patients with hypertension, we conducted this two‐center prospective longitudinal cohort study.

## 2 METHODS

### 2.1 Patients and clinical data

This is a prospective longitudinal observational study including patients from two centers (West China Hospital of Sichuan university and Chengdu 7th People's Hospital) in China. Adult patients were eligible if they: (1) had chronic kidney disease with stage 1‐4;^18^ (2) have been diagnosed with hypertension; and (3) agreed to take 24h ABPM. Exclusion criteria were patients with (1) end‐stage renal disease (eGFR≤15 mL/min per 1.73 m^2^), dialysis or kidney transplant; (2) history of malignancy. Patients with albuminuria (albumin/creatinine ratio ≥30 mg/g), or the estimated glomerular filtration rate (eGFR) < 60 mL/min /1.73 m^2^, or abnormalities of kidney structure for over 3 months were diagnosed with CKD.^18^ The eGFR value was estimated from serum creatinine levels with Chronic Kidney Disease Epidemiology Collaboration (CKD‐EPI) equation.^19^ The study protocol was approved by the ethics committee of the West China hospital, Sichuan University, and was approved by the Institutional Review Board (http://www.thaiclinicaltrials.org/; TCTR20180313004). Written informed consent was obtained from all patients.

Enrollment started in January 2014 and a total of 304 CKD patients met the inclusion /exclusion criteria and completed the 24h ABPM by December 2019. Demographic data (age, gender, BMI), Medical history (current alcohol, current smoking, CV history, antihypertensive drugs, diabetes mellitus (DM)), and laboratory tests (biochemical parameters, urinary protein test) were recorded at baseline.

### 2.2 Blood pressure measurements

Office blood pressure was measured by experienced nurses at the time that the participants admitted to our hospital or went to outpatient clinic, the mean value of three consecutive measurements at 5‐min intervals with a mercury sphygmomanometer after patients rest quietly for 5–10 min was recorded. The 24h ABPM was performed via The Space Labs 90217 device (Space Labs Medical, Redmond, Washington, USA) in West China Hospital and ABPM 6100 (Welch Allyn, USA) in Chengdu 7th People's Hospital, with BP readings set at 20‐min intervals from 6:00 am to 10:00pm and 30‐min or 60‐min intervals from 10:00pm to 6:00am. The BP and heart rate (HR) of day‐time, nighttime were defined as the mean values during the period from 6:00am to 10:00pm and 10:00pm to 6:00am, respectively; patients were instructed to take their usual activities and receive antihypertensive drugs as usual, and was encouraged to sleep no later than 10:00pm, and get up at nearly 6:00am. A measurement with at least 70% of diurnal and nocturnal BP readings was regarded as a successful ambulatory BP. Both office BP and ambulatory BP measurements were taken from the nondominant arm with an appropriate cuff size based on arm circumference at the time of enrollment.

### 2.3 Definitions

Sleep‐trough MBPS was defined as the average of systolic BP (SBP) during the 2 h immediately after awakening minus the average of the 3 SBP readings centered around the lowest night SBP value^20^; patients were divided into MBPS group (≥ 15 mmHg) and non‐ MBPS group (< 15 mmHg) according to third quantile. Dipping pattern of BP and HR were calculated with the formula: mean night/ day ratio of SBP, diastolic BP (DBP) and HR, patients were diagnosed with normal dippers if the ratio was > 0.8 to 0.9, extreme dippers if the ratio < 0.8, non‐dippers if the ratio > 0.9 to 1, or reverse dipper if the ratio > 1.^21^ Patients were defined to have achieved the goal for ambulatory BP when 24‐h, daytime, and nighttime BP was < 130/80, < 135/85, and < 120/70 mmHg, respectively, and to have achieved the goal for office BP when BP was < 140/90.^22^, ^23^ Morning hypertension is defined as the mean BP within 2 h after awakening ≥135/85 mmHg .^24^ Left ventricular hypertrophy (LVH) was defined according to the 2015 American Society Echocardiography (ASE)/European Association of Cardiovascular Imaging (EACVI) chamber quantification document, with the LV mass index (LVMI) by body surface area > 95 g/ m^2^ in women and 115 g/ m^2^ in men.^25^

### 2.4 Outcomes

The primary outcome was CKD progression, which was defined as a composite of progression to ESRD, or eGFR decline ≥ 50%. The secondary outcome included (1) death; (2) CV events, which defined as a fatal or nonfatal newly occurred CV disease, including coronary heart disease, myocardial infarction, heart failure, stroke, angina pectoris, whichever occurred first. Patients were followed up by checking the electronic databases in the hospital and phone calls; the follow up was from the day of ABPM, until December 2020, death, or CKD progression, and censored on the date they had the last clinic visit or phone answering.

### 2.5 Statistical analysis

Statistical analysis was performed using IBM SPSS Statistics for Windows, Version 20.0 (IBM Corp, Armonk, NY, USA). All data were expressed as mean ± standard deviation (SD) for normally distributed data, median values with their interquartile range for skewed data and numbers (n) with percentage (%) for categorical variables. Data were analyzed by using the Chi‐square test or Fisher's exact test for categorical variables, Student's *t*‐test for normally distributed data, and Wilcoxon rank sum test for continuous skewed variables.

Receiver‐operating characteristic (ROC) curve analysis was conducted, and the area under the curve (AUC) was calculated to understand the ability of morning BP and night BP to distinguish elevated MBPS. Binary logistic regression was applied to detect the factors associated with elevated MBPS. Kaplan‐Meier survival function estimates, log‐rank test was applied to compare the incidence rates of events in different groups; Multivariable Cox regression model was used to investigate associations of MBPS with death, renal and CV outcomes; six models were conducted, baseline demographic characteristics (age, gender, BMI, etc.) and baseline biochemical parameters including hemoglobin, platelet, albumin, creatinine were adjusted in model 2 and 3, while some interesting ABPM indices were further adjusted in model 4‐6, involving SBP and HR of day, night, and morning, HR dipping (model 4), BP control of morning and night, HR dipping (model 5), and control of office and ambulatory BP, HR dipping (model 6). The proportional hazards (PH) assumption was tested by assessing the log‐minus‐log plots of survival. Hazard Ratios (HRs), 95% confidence intervals (CIs) were calculated and a two‐tailed *p* value < .05 was considered statistically significant.

## 3 RESULTS

### 3.1 Baseline characteristics

In this two‐center prospective observational cohort study, a total of 304 CKD patients with hypertension were enrolled (210 with non‐MBPS and 94 with MBPS). The baseline characteristics of the patients were listed in Table 1. The mean age was 72 ±11 years, 133 (43.8%) were male. The number of patients with current smoking, current alcohol consumption. CV history, diabetes mellitus, diabetic kidney disease and left ventricular hypertrophy were 48 (15.8%), 46 (15.1%), 123 (40.5%), 219 (72.0%), 172 (56.6%), and 61 (20.1%), respectively. Most patients had nocturnal hypertension (65%), and non‐dipping BP pattern (90.5%). The mean MBPS was 9.25 ±0.84 mmHg, the third quantile was 15 mmHg.

**TABLE 1:** Demographic and clinical characteristics between low morning surge and elevated morning surge group

Variables	Non‐ MBPS group (No. = 210)	MBPS group (No. = 94)	*p*
Age (years)	72.18±11.04	71.10±9.49	.121
Male (no., %)	90 (42.9)	43 (45.7)	.639
BMI (kg/m^2^)	24.89±4.51	24.39±3.99	.366
Smoking (no., %)	33 (15.7)	15 (16.0)	.957
Alcohol (no., %)	29 (13.8)	17 (18.1)	.336
CV history (no., %)	91 (43.3)	32 (34.0)	.127
DM (no., %)	150 (71.4)	69 (73.4)	.723
DKD (no., %)	122 (58.1)	50 (53.2)	.425
CCB (no., %)	130 (61.9)	58 (61.7)	.973
RAS (no., %))	110 (52.4)	41 (43.6)	.158
Diuretic (no., %)	31 (14.8)	9 (9.6)	.216
β blocker (no., %)	71 (33.8)	26 (27.7)	.288
α blocker (no., %)	1 (0.5)	4 (4.3)	.033
Numbers of drugs (> 2) (no., %)	43 (20.5)	13 (13.8)	.167
LVH (no., %)	40 (19.0)	21 (22.3)	.508
Red blood cells (10^12^/L)	4.14±0.71	4.12±0.69	.894
Hemoglobin (g/L)	122.71±21.04	121.86±19.94	.742
Platelet (10^9^/L)	154 (117.8, 207)	169 (136.8, 225.3)	.012
White blood cells (10^9^/L)	6.10 (4.76, 7.59)	6.44 (5.21, 7.61)	.351
Total protein (g/L)	68.30±7.06	69.60±6.88	.135
Albumin (g/L)	39.15 (35.90, 42.43)	39.95 (37.08, 42.83)	.186
Creatinine (umol/L)	104.0 (87.6, 130.1)	117.3 (97.7, 136.8)	.009
Uric acid (μmol/L)	360.2±112.5	399.1±127.2	.004
Cystatin C (mg/L)	1.02 (0.78, 1.51)	1.08 (0.80, 1.68)	.508
eGFR (mL/min per 1.73 m^2^)	51.0 (39.5, 62.4)	48.0 (36.5, 58.3)	.072
Kalium (mmol/L)	3.99 (3.68, 4.32)	4.10 (3.82, 4.38)	.063
Sodium (mmol/L)	139.85 (137.10, 142.60)	140.75 (138.17, 142.75)	.172
Chlorine (mmol/L)	106.5 (103.2, 109.8)	107.9 (104.8, 110.9)	.03
Calcium (mmol/L)	2.31±0.18	2.33±0.16	.465
Phosphorus (mmol/L)	1.05 (0.92, 1.19)	1.05 (0.91, 1.18)	.671
Magnesium (mmol/L)	0.86±0.11	0.86±0.10	.951
Cholesterol (mmol/L)	4.37 (3.63, 5.18)	4.46 (3.65, 5.15)	.944
Triglyceride (mmol/L)	1.33 (0.92, 2.27)	1.39 (0.87, 2.24)	.924
High density lipoprotein (mmol/L)	1.15 (0.96, 1.48)	1.22 (0.94, 1.56)	.472
Low density lipoprotein (mmol/L)	2.89±1.05	2.83±1.10	.668
Left ventricular mass index (g/ m^2^)	81.90 (72.45, 100.71)	86.52 (73.34, 98.04)	.606
UACR	89.60 (38.00, 249.22)	108.46 (36.61, 359.85)	.495

Abbreviations: CCB, calcium channel blocker; CKD, chronic kidney disease. UACR, Urinary microalbumin/creatinine ratio; CV, cardiovascular; DKD, diabetic kidney disease; DM, diabetes mellitus; eGFR, estimated glomerular filtration rate; LVH, left ventricular hypertrophy; MBPS, morning blood pressure surge. BMI, body mass index; RAS, renin‐ angiotensin system.

In contrast to patients with non‐ MBPS (< 15 mmHg), those with MBPS (≥ 15 mmHg) had higher level of platelet, creatinine, uric acid and chlorine and lower level of eGFR. Besides, the percentage of using α blocker was also higher in patients with MBPS ≥ 15 mmHg (*p* = .033), the details are shown in Table 1.

As for the ABPM outcome, a lower level of SBP dipping, DBP dipping, and HR dipping was found in MBPS group (Table 2). Besides, in the MBPS group, there was a poorer control of morning BP (*p* < .001) compared with non‐ MBPS group, while the control of office, 24 h, day and night BP demonstrated no differences (Table 2).

**TABLE 2:** The blood pressure characteristics from ABPM between non‐MBPS and MBPS group

Variables	Non‐ MBPS group (no. = 210)	MBPS group (no. = 94)	*p*
Office SBP (mmHg)	142.99±21.23	141.90±18.13	.668
Office DBP (mmHg)	80.00±12.04	78.17±11.71	.218
Office BP control (no., %)	89 (42.4)	40 (42.6)	.978
24h			
SBP (mmHg)	127.84±15.39	128.31±14.32	.802
DBP (mmHg)	65.80±9.49	67.51±9.48	.147
HR (bpm)	72±10	74±10	.026
24h BP control (no., %)	119 (56.7)	49 (52.1)	.462
Day			
SBP (mmHg)	127.83±15.64	129.81±14.68	.300
DBP (mmHg)	65 (58.8, 72.0)	68 (62.0, 74.3)	.025
HR	72±10	76±11	.007
Day BP control (no., %)	139 (66.2)	54 (57.4)	.143
Night			
SBP (mmHg)	128.5±17.3	124.1±15.3	.032
DBP (mmHg)	64.0 (57.8, 71.0)	62.5 (57.0, 69.0)	.402
HR	66 (61, 73)	68 (62, 76)	.167
Night BP control (no., %)	69 (32.9)	38 (40.4)	.004
SBP dipping	1.01±0.07	0.96±0.07	*p* < .001
DBP dipping	0.98±0.83	0.93±0.07	*p* < .001
HR dipping	0.94±0.08	0.92±0.08	.039
SBP dipping patterns (no., %)			*p* < .001
Dippers	9 (4.3)	20 (21.3)	
Non‐dippers	98 (46.7)	53 (56.4)	
Reverse dippers	103 (49)	21 (22.3)	
DBP dipping patterns (no., %)			*p* < .001
Dippers	25 (11.9)	25 (26.6)	
Non‐dippers	106 (50.5)	46 (48.9%)	
Reverse dippers	77 (36.7)	19 (20.2)	
Extreme dippers	2 (1)	4 (4.3)	
ABP control (no., %)	69 (32.9)	37 (39.4)	.271
Morning SBP (mmHg)	126.2±17.0	139.8±17.5	*p* < .001
Morning DBP (mmHg)	66.8±11.1	74±11.5	*p* < .001
Morning BP control (no., %)	151 (71.9)	44 (46.8)	*p* < .001

Abbreviations: BP, blood pressure, HR, heart rate.; DBP, diastolic blood pressure; MBPS, morning blood pressure surge; ABPM, ambulatory blood pressure monitoring; SBP, systolic blood pressure.

### 3.2 Blood pressure indices differentiating elevated MBPS

**FIGURE 1:**
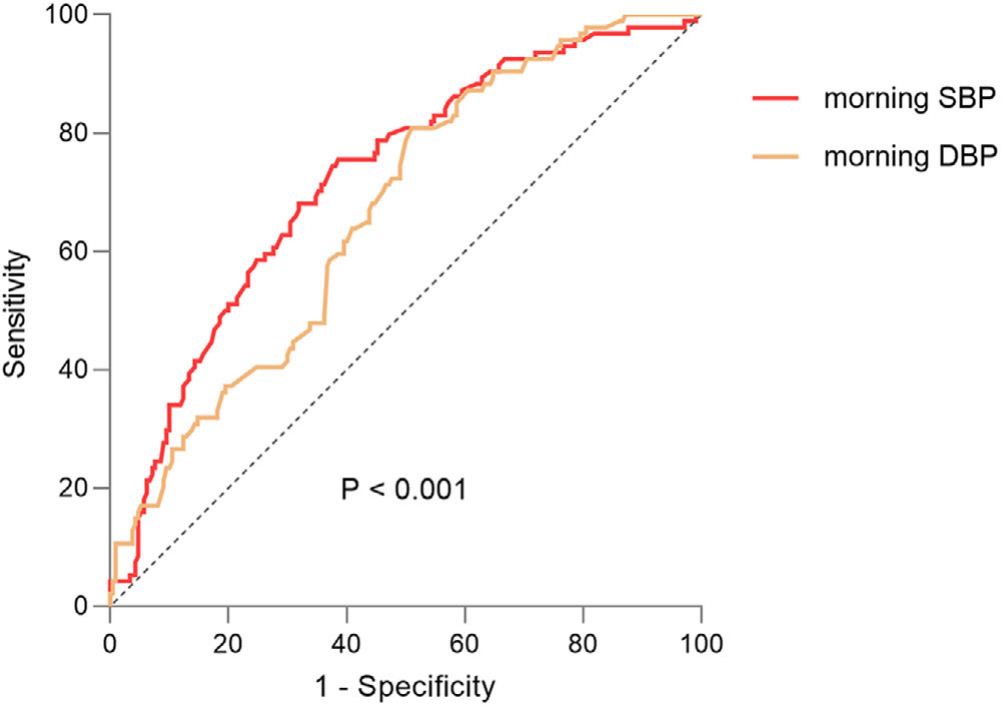
The ROC curves of morning SBP and DBP about elevated MBPS. Abbreviations: ROC, Receiver‐operating characteristic; SBP, systolic blood pressure; DBP, diastolic blood pressure

The ROC curve analysis was conducted using MBPS as a state variable indicating elevated MBPS (≥ 15 mmHg), and using SBP and DBP of morning and night as test variables (Figure 1). The analysis revealed morning SBP (AUROC 0.723, 95%CI 0.66‐0.78, *p* < .001) and DBP (AUROC 0.67, 95%CI 0.61‐0.73, *p* < .001) could distinguish elevated MBPS with the optimal cut‐off points of 129/65 mmHg, whereas night SBP and DBP could not (data not shown).

### 3.3 Incidence of events and survival analysis

During the follow‐up period of 30 (interquartile range: 19–48) months, two participants were excluded from the study because of lost to follow up. Finally, 302 participants (208 in non‐ MBPS group and 94 in MBPS group) were included into the analysis. In MBPS group, 18 (19.1%), 31 (33%) patients occurred CKD progression and CV events, while the number was 16 (7.6%), 64 (30.5%) in non‐ MBPS group. The mortality was 8.5% (8 in 94 patients, including 4 for CV events, 2 for ESRD, 1 for infection, 1 for cancer) in MBPS group, and 7.1% (15 in 210 patients, including 4 for cancer, 5 for severe infection, 2 for ESRD, 2 for CV events, and 2 for gastrointestinal hemorrhage) in non‐MBPS group.

**FIGURE 2:**
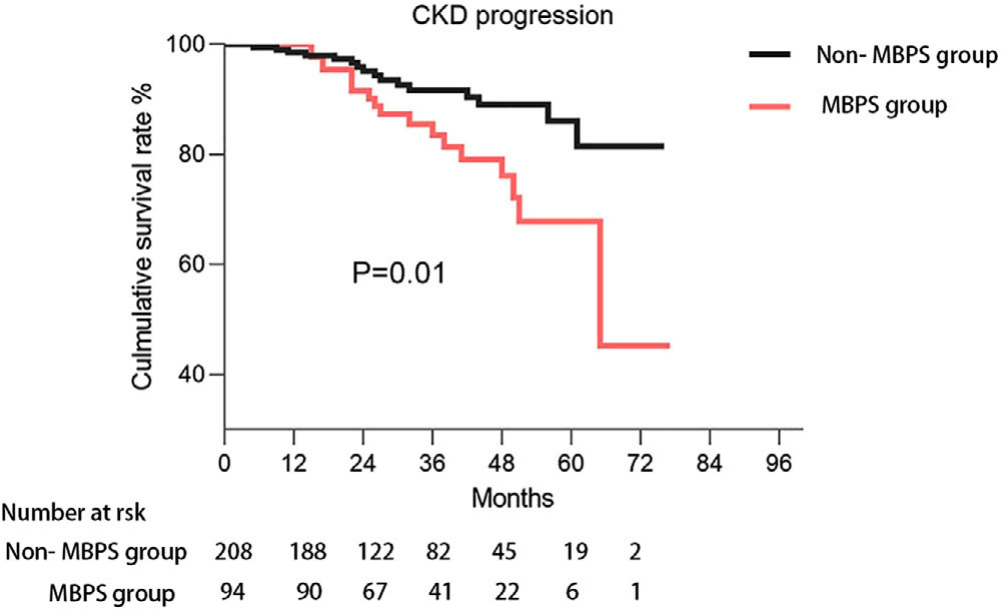
Kaplan‐Meier hazard curve for CKD progression by non‐MBPS and MBPS group. Abbreviations: CKD, chronic kidney disease; MBPS, morning blood pressure surge

As shown in Figure 2, cumulative survival rate for CKD progression was significantly lower in patients with MBPS group (*p* = .01), while there were no differences in death and CV events (data not shown). In the Cox regression model, MBPS was associated with a higher risk of CKD progression (HR 2.35, 95%CI 1.20‐4.63, *p* = .013, Table 3), the difference remained significant even after adjustment for baseline demographic characteristics and some biochemical parameters (Model 2 and model 3). Notably, the predictive value was also independent of day, night BP (RR 2.75, 95%CI 1.03‐7.34, *p* = .043, model 4), night and morning BP control (RR 2.36, 95%CI 1.09 ‐5.12, *p* = .03, model 5), and office, ambulatory BP control (RR 3.23, 95%CI 1.31‐7.95, *p* = .039, model 6). The association of MBPS with CV events (HR 1.02, 95%CI 0.66‐1.57) and death (HR 1.08, 95%CI 0.46‐2.55) were also analyzed, no predictable value was found (Table 3).

**TABLE 3:** Multivariate Cox regression analysis of the association of elevated morning surge with CKD progression, CV events, and death

Variables	Model 1	Model 2	Model 3	Model 4	Model 5	Model 6
CKD progression						
Non‐ MBPS group (< = 15 mmHg)	Reference	Reference	Reference	Reference	Reference	Reference
MBPS group (> 15 mmHg)	2.35 (1.20, 4.63) ^*^	2.26 (1.13, 4.51) ^*^	2.29 (1.10, 4.77) ^*^	2.75 (1.03, 7.34) ^*^	2.36 (1.09, 5.12) ^*^	2.26 (1.04, 4.89) ^*^
CV events						
Non‐ MBPS group (< = 15 mmHg)	Reference	Reference	Reference	–	–	–
MBPS group (> 15 mmHg)	1.02 (0.66, 1.57)	0.97 (0.63, 1.51)	0.92 (0.59, 1.44)	–	–	–
Mortality						
Non‐ MBPS group (< = 15 mmHg)	Reference	Reference	Reference	–	–	–
MBPS group (> 15 mmHg)	1.08 (0.46, 2.55)	1.24 (0.52, 3.02)	1.31 (0.53, 3.26)	–	–	–

Model 1: unadjusted.

Model 2: 1+gender, age, BMI, diabetes mellitus, CV events, RAS inhibitors.

Model 3: model 2 + hemoglobin, platelet, albumin, creatinine.

Model 4: model 3 +day SBP, day HR, night SBP, night HR, morning SBP, HR dipping.

Model 5: model 3 +morning BP control, night BP control, HR dipping.

Model 6: model 3+office BP control, ABP control, HR dipping.

Abbreviations: CV, cardiovascular; MBPS, morning blood pressure surge; CKD, chronic kidney disease; RAS, renin‐angiotensin system. *p < .05.

## 4 DISCUSSION

In this prospective longitudinal cohort study, we firstly investigated the relationship between sleep‐trough MBPS and prognosis in CKD stage 1–4 patients with hypertension. In these patients, the MBPS was much lower than that reported previously, but elevated MBPS also showed a significant association with CKD progression independent of morning BP, BP dipping and control of ambulatory BP and office BP. Conversely, the elevated MBPS was not found to be correlated with death and CV events. These data suggests that elevated MBPS plays an important role in CKD progression in CKD patients with hypertension.

The existing literature have investigated the potential impact of MBPS on impaired renal function and the development of CKD. For example, a prospective cohort study confirmed the correlation between MBPS and decline in eGFR (β −0.549, 95%CI −0.750 ‐ −0.349) and incident CKD (HR 1.718, 95%CI 1.121‐2.635) in 622 hypertensive patients.^15^ Among 823 CKD patients, a cross‐sectional study showed significant evidence of excessive MBPS was an independent factor strongly related to poor renal function (β −0.094, 95%CI −0.180 ‐ −0.007).^14^ Our study has some differences. First, we studied the association of elevated MBPS with CKD progression (progression to ESRD, or decline in eGFR ≥ 50%), rather than the change of eGFR,^15^ which had a large significance in the clinic practice; Second, all patients were complicated with hypertension and CKD, and most patients had nocturnal hypertension (65%) or non‐dipping BP pattern (90.5%); under this situation, MBPS is much lower than that reported by previous studies,^14^, ^15^ but elevated MBPS also showed associations with CKD progression in our study, regardless of morning BP control; this revealed the necessity and importance of appropriate evaluation and management of MBPS in patients with CKD and hypertension.

Morning BP and MBPS are distinct but related BP indices, elevated MBPS was often accompanied with high morning BP, our study also showed their close connection. Morning BP could distinguish elevated MBPS well ((AUROC 0.723, 95%CI 0.66‐0.78, *p* < .001) (Figure 1), the optimal morning SBP cut‐off for detecting abnormal MBPS was 129 mmHg; furthermore, 53.2% patients in the elevated MBPS group had morning hypertension, versus 28.1% in low MBPS group (Table 2); Therefore, our findings suggest that it is possible to detect abnormally elevated MBPS by monitoring home morning BP if the ABPM was not available.

Although the intimate correlation between morning BP and MBPS existed, and morning hypertension has been reported to be a risk factor of declined kidney function,^26^, ^27^ the predictive value of elevated MBPS to CKD progression was independent of morning BP, as well as BP dipping and ambulatory BP/ office BP control, indicating that MBPS held some independent characteristics which were associated with CKD progression. It has previously been observed that elevated MBPS was related to urinary albumin excretion and albumin/creatinine ratio .^28^ Albuminuria is an established sign of kidney damage, and indicates an increased risk of kidney and CV disease because of insulin resistance and endothelial dysfunction.^29^ Based on present evidences, it is speculated that elevated MBPS rises the glomerular pressure load rapidly in a short time and injures renal vascular endothelium, resulting in poor renal function.^14^ Previous studies also reported that elevated MBPS was associated with impaired baroreceptor sensitivity, neurohormonal abnormalities of the renin‐angiotensin system and sympathetic nervous system, these factors contribute to vascular damage from the larger arteries to the small resistance vessel^30^, ^31^; furthermore, as Komori and associates demonstrated, elevated MBPS can induce increased afterload, thus results in decreased stroke volume.^31^ These cardiovascular damages may affect renal blood perfusion, which is likely to contribute to the CKD progression. However, the underlying mechanisms regarding MBPS and CKD progression are still unclear, further studies are needed.

In this study, we also analyzed the association of elevated MBPS with CV events and death. Elevated MBPS was not found to be an independent predictor of CV events and death, consistent with some previous studies. An analysis of 2051 subjects indicated a weak positive relationship between elevated MBPS and CV events or death, which disappeared after adjustments.^32^ Another study in blacks showed that MBPS itself was not associated with increased CV risk and death.^33^ However, many studies reported the predictive value of MBPS to CV events in non‐CKD patients.^34^, ^35^, ^36^ In a systematic meta‐analysis covering 5645 patients, elevated MBPS (≥37 mmHg) was demonstrated to predict independently CV events(HR 1.30, 95%CI 1.06‐1.60) and death (HR 1.32, 95%CI 1.09‐1.59).^37^ The contrary conclusions can be attributed to a different classification of elevated MBPS. As we mentioned above, 65% of the patients enrolled in this study had nocturnal hypertension and 90.5% had non‐dipping BP pattern, so elevated MBPS in this study was defined as ≥15 mmHg while others were defined as ≥25 mmHg or higher^35^, ^37^; a MBPS in SBP < 20 mmHg was probably not associated with an increased risk of death or cardiovascular events.^37^ In addition, the differences of study population may be another reason. The study population in this study were patients with stage 1–4 CKD and hypertension while previous studies were mostly hypertensives without CKD.

Our study has some strengths. First, to our knowledge, this is the first prospective study to investigate clinical predictive value of elevated MBPS to CKD progression in patients with CKD and hypertension. Second, this study has high rates of follow‐up. There are also some limitations. Only 304 patients were included into this study, the sample is relatively small. And the median follow‐up time is 2.5‐year, which is not long enough. A larger sample size prospective study with enough follow‐up time is needed in the future. Besides, our data were from Chinese population, so it is not sure whether these findings are appropriate for other ethnic groups. Therefore, other validation enrolling other ethnics would be required.

In conclusion, we provided the first evidence that elevated MBPS was an important risk factor of CKD progression in patients with CKD and hypertension. Appropriate evaluation and management of MBPS may be helpful to postpone CKD progression.

## CONFLICTS OF INTEREST

The authors declared no potential conflicts of interest concerning the research, authorship, and/ or publication of this article.

## AUTHOR CONTRIBUTIONS

Xiang Liu, Ting Zhang, Yi Tang, and Wei Qin: Substantial contributions to the conception or design of the work; or the acquisition, analysis, or interpretation of data for the work; AND drafting the work or revising it critically for important intellectual content; AND final approval of the version to be published; AND agreement to be accountable for all aspects of the work in ensuring that questions related to the accuracy or integrity of any part of the work are appropriately investigated and resolved.

Fangming Li, Zhiyao Zheng, and Huan Zhou, Aiya Qin: analysis and acquisition of data; AND drafting the work; AND final approval of the version to be published; AND agreement to be accountable for all aspects of the work in ensuring that questions related to the accuracy or integrity of any part of the work are appropriately investigated and resolved.
